# Antibiotic resistance profiles of gut microbiota across various primate species in Guangxi

**DOI:** 10.3389/fmicb.2023.1309709

**Published:** 2023-12-14

**Authors:** Hongli Huang, Xianwu Pang, Tengcheng Que, Panyu Chen, Shousheng Li, Aiqiong Wu, Meihong He, Hong Qiu, Yanling Hu

**Affiliations:** ^1^Clinical Biological Specimen Bank, Discipline Construction Office, The First Affiliated Hospital of Guangxi Medical University, Nanning, Guangxi, China; ^2^Guangxi Zhuang Autonomous Region Center for Disease Prevention and Control, Nanning, Guangxi, China; ^3^Faculty of Data Science, City University of Macau, Macau SAR, China; ^4^Right River National Medical College, Baise, Guangxi, China; ^5^Guangxi Zhuang Autonomous Region Terrestrial Wildlife Course Research and Epidemic Diseases Monitor Center, Nanning, Guangxi, China; ^6^Life Sciences Institute, Guangxi Medical University, Nanning, Guangxi, China; ^7^Department of Biochemistry and Molecular Biology, School of Pre-Clinical Medicine, Guangxi Medical University, Nanning, Guangxi, China; ^8^Center for Genomic and Personalized Medicine, Guangxi key Laboratory for Genomic and Personalized Medicine, Guangxi Collaborative Innovation Center for Genomic and Personalized Medicine, Guangxi Medical University, Nanning, Guangxi, China

**Keywords:** antibiotic resistance genes, resistome, gut microbiota, non-human primates, metagenomics

## Abstract

**Introduction:**

Understanding the gut microbiota and antibiotic resistance gene (ARG) profiles in non-human primates (NHPs) is crucial for evaluating their potential impact on human health and the environment.

**Methods:**

In this study, we performed metagenomic analysis of 203 primate fecal samples, including nine NHP species and humans, to comprehensively characterize their gut microbiota and ARGs.

**Results:**

Our study reveals the prevailing phyla in primates as Firmicutes, Bacteroidetes, Euryarchaeota, and Proteobacteria. The captive NHPs exhibited higher ARG abundance compared to their wild counterparts, with tetracycline and beta-lactam resistance genes prevailing. Notably, ARG subtypes in Trachypithecus leucocephalus (T. leucocephalus) residing in karst limestone habitats displayed a more dispersed distribution compared to other species. Interestingly, ARG profiles of NHPs clustered based on geographic location and captivity status. Co-occurrence network analysis revealed intricate correlations between ARG subtypes and bacterial taxa. Procrustes analysis unveiled a significant correlation between ARGs and microbial phylogenetic community structure. Taxonomic composition analysis further highlighted differences in microbial abundance among NHPs and humans.

**Discussion:**

Our study underscores the impact of lifestyle and geographical location on NHP gut microbiota and ARGs, providing essential insights into the potential risks posed by NHPs to antibiotic resistance dissemination. This comprehensive analysis enhances our understanding of the interplay between NHPs and the gut resistome, offering a critical reference for future research on antibiotic resistance and host-microbe interactions.

## 1 Introduction

Antibiotic resistance is a significant global threat (Bolan et al., 2012; Brinkac et al., 2017; Qiao et al., 2018; Gil-Gil et al., 2021; Mustafa et al., 2021). More than 100,000 tons of antibiotics are produced worldwide each year and are extensively used in aquaculture, healthcare, and clinical laboratories, leading to widespread drug resistance in many bacteria and the emergence of ARGs (van Boeckel et al., 2015; Zhang et al., 2015; Xu et al., 2017; Sun et al., 2019; Wang et al., 2019; Qiu et al., 2020). The World Bank has warned that if we do not take strong actions, antimicrobial resistance is expected to kill 10 million people by 2050, with accumulative economic losses reaching 100 trillion dollars (He et al., 2019; Qian et al., 2021). What’s worse, ARGs are widely distributed in sewage, soil, surface water, medical waste, and animal feces, and they can be transmitted to humans through agriculture products, water, or air in both captive and wild environments, which will increase the difficulty of dealing with antibiotic resistance (Chi et al., 2020; Yang et al., 2022). From the perspective of “One Health,” an effective way to accelerate harmless and resourceful treatment of ARGs is crucial (Su et al., 2023).

NHPs are genetically and evolutional relatives of humans, and research has shown that the gut microbiome of them is biologically similar to humans (Yildirim et al., 2010). It was once believed that humans evolved from apes, so NHP would be an excellent model for studying the spread and prevention of ARGs. At present, there are many kinds of NHPs lives in Guangxi, China, including *Trachypithecus leucocephalus* (*T. leucocephalus*), *Trachypithecus francoisi* (*T. francoisi*), *Macaca multta* (*M. multta*), etc., which is reliable resources for the study of non-human primates. Studies on them have focused on genome adaptation, conservation, and behavior (Huang and Li, 2005; Li et al., 2020; Liu Z. J. et al., 2020; Fan and Pedersen, 2021; Que et al., 2021), but little is about the diversity and composition of ARGs. Identification of ARGs in NHPs can be used as a proactive method for monitoring antibiotic resistance and as a control measure against infection (Schuchat et al., 2001; Roca et al., 2015; Liu et al., 2016). A study showed that seasonal variations in the gut microbiota of free-range white-faced capuchins, but their ARGs composition remained unknown (Orkin et al., 2019). The composition and abundance of ARGs carried by the gut microbiota of cynomolgus monkeys were significantly affected by dietary changes (Yan et al., 2022). Some antibiotics, such as beta-lactam, aminoglycoside, macrolides, and bacitracin in captive pandas were significantly higher than those in wild pandas (Guo et al., 2019). Nevertheless, few studies have been conducted on the gut microbiota of multiple NHPs, and their gut microbiota and composition of the ARGs they carried are unknown. Therefore, there is an urgent need to fully understand the gut microbiota of NHPs and the composition of ARGs carried by NHPs, especially in the endangered primate *T. leucocephalus*.

Karst limestone is one of the typical geographical features of Guangxi, and it is the habitat of *T. leucocephalus*. Whether karst limestone has an impact on the gut microbiota and ARGs of primates, including *T. leucocephalus,* remains to be further investigated. Our previous findings showed that the gut microbiota of *T. leucocephalus* is mainly related to heat tolerance and cellulose degradation. Xiang et al. (2020) found that the diversity, and composition of microbiota in karst water in southwest China were closely related to ARGs and antibiotic usage. Stange and Tiehm (2020) detected beta-lactams, aminoglycosides, tetracyclines, macrolides, and beta-lactams in karst lava springs in Germany, the frequency of *erm*B (42.1%), *tet* (C) (40.8%), *sul*2 (39.5%), and s*ul*1 (36.8%) was higher. With increasing detection levels of ARGs detected in the environment, we speculate that the karst limestone in Guangxi has an impact on the gut microbiota and ARGs of local primates, and they play an important role in the development and human-to-human transmission of ARGs (Allen et al., 2010).

To uncover these, 150 fecal samples of 9 NHPs were collected in Guangxi. At the same time, metagenomic data of Chinese population was downloaded from the database. In this study, high-throughput sequencing and metagenomic analysis of ARGs in NHPs in Guangxi was conducted to explore the driving factors of lifestyle, geographical location and host species on ARGs, and to study the symbiotic network between ARG subtypes and related microbiota. This study will provide a comprehensive understanding of the distribution of ARGs in the NHPs gut microbiota in Guangxi and reveal their potential risks to human health.

## 2 Methods

### 2.1 Sample collection and public datasets acquisition

A total of 150 fecal samples were collected, including 27 *T. leucocephalus* (1 from Terrestrial Wildlife Course Research and Epidemic Diseases Monitor Center and 26 from Chongzuo White-headed Langur National Nature Reserve), 20 *T. francoisi* (6 from Wuzhou Langur Breeding and Research Center and 14 from Nanning Zoon), 5 *Papio*, 5 *Hylobatidae*, 9 *Trachypithecus cristatus* (*T. cristatus*), 23 *Lemur catta* (from Nanning Zoon), 8 *Nycticebus pygmaeus* (*N. pygmaeus*), 19 *Nycticebus coucang* (*N. coucang*) (from Terrestrial Wildlife Medical-aid Monitoring Epidemic Diseases Research Center), and 34 *M. mulatta* samples (from Hezhou Gupo Mountain) (Supplementary Table S1; Supplementary Figure S1). All the samples were collected during the spring of 2018 to mitigate potential seasonal variations in microbial content.

To obtain wild fecal samples, we strategically placed food on the ground and concealed ourselves in vegetation. Upon defecation, we used latex gloves to select fresh samples based on visual cues related to sample integrity and color. For captive specimens, healthy NHPs were randomly chosen, and their fecal samples were promptly collected in an aseptic manner post-defecation. Using a spoon, feces from the center portion were collected and deposited into sterile containers, which were then transported to the laboratory in a low-temperature box accompanied by dry ice. Subsequently, samples were immediately frozen and stored at −80°C for subsequent processing. DNA was extracted within 48 h following samples delivery. The research adhered to protocols established by the China Wildlife Conservation Association and the legal requirements of China.

For comparative analysis, metagenomic data (PRJEB21612) of 53 health humans in China were downloaded from the European Nucleotide Archive Database.^1^ These datasets were incorporated into our study to enhance the breadth of our analysis.

### 2.2 DNA extraction and sequencing

DNA extraction was conducted from 200 mg frozen fecal samples using the Qiagen DNA extraction kit (Qiagen, Germany) according to the manufacturer’s instructions. DNA concentration and purity were assessed using the Qubit® 2.0 Flurometer (Life Technologies, CA, USA) and NanoPhotometer (IMPLEN, CA, USA), respectively. DNA integrity was evaluated through 1% agarose gel electrophoresis. Metagenomic DNA was fragmented using ultrasonic Processor, yielding an average size of approximately 350 bp. A paired-end library was generated using the TruSeq DNA Sample Preparation Guide (Illumina, 15,026,486 Rev.C). High-throughput paired-end sequencing was performed on the Illumina HiSeq X-ten platform (Illumina, USA) utilizing the index PE150 sequencing strategy. Subsequently, sequences with low-quality bases (Q ≤ 19) at the terminal regions were trimmed to ensure high-quality data. Reads containing substantial “N” (≥5% of the read), adapter contamination, or those falling below a specified length (>150-bp) were filtered out. Following these steps, a set of clean data possessing high quality were obtained for further analysis.

### 2.3 Profiling of ARGs and microorganism composition

We quantified the profiles of ARGs in the 203 metagenomic datasets using the ARGs-OAP pipeline (version 2.0) (Feng et al., 2018; Yin et al., 2018; Yang et al., 2019; Qian et al., 2021).^2^ The datasets were aligned to a structured ARG (SARG) database following established protocols outlined in prior publications. Specifically, we combined the metadata file of samples with the clean reads files in a single directory within our local Linux system. The initial Perl script was executed to identify ARG-like sequences and 16S ribosomal RNA (rRNA) gene sequences, with parameters set at an alignment length of 25 amino acids, 80% sequence similarity, and an e-value of 1 × 10^−7^ (Feng et al., 2018; Yin et al., 2018). Subsequently, a candidate ARG sequences file and an online metadata file were matched against the integrated structure to align with SARG reference databases, encompassing 24 types and 1,244 subtypes of ARGs, using the second Perl script within our local Linux system. ARG abundances are normalized with respect to total 16S reads (unit: copies of ARG per copy of 16S rRNA), the number of cells (unit: ARG copies per cell), and ppm (reads carrying ARGs per million reads), a unit normalized by dividing the total reads carrying ARGs by a million read counts (Zheng et al., 2022). This process yielded the ARG profile abundance at different levels, type, subtype level, and gene (reference sequence).

For taxonomic composition analysis, metagenomes data underwent taxonomic classification and relative abundance quantification (in terms of the number of cells, not just fraction of reads) through HUMAnN2 pipeline (v0.11.1) integrated with the MetaPhlAn database of marker genes mpa_v20_m200 with settings (Li et al., 2015; Gacesa et al., 2022). Differential abundances of taxa were identified using LDA effect size (LEfSe) (Yang et al., 2022).

### 2.4 Procrustes analysis

We conducted correlation analyses between ARGs-ARGs and ARGs-genus taxa using ‘vegan’ package in R (v.4.3.1) (Yang et al., 2022). For comparisons of *M*^2^ values with 999 permutations, Spearman’s correlation coefficients were calculated using the protest function (Qian et al., 2021). The *M*^2^ value, representing the sum of the squared distances between matched sample pairs, was computed. To obtain the *p*-value, the original *M*^2^ value was compared to the simulated distribution of *M*^2^ values. When the *p*-value was below 0.05, a significant correlation between antibiotic resistance and bacterial species was established (Feng et al., 2018).

### 2.5 Statistical and network analysis

Principal coordination analysis (PCoA) and principal component analysis (PCA) were conducted base on the ARG subtype and taxonomic abundance tables. A heat map illustrating the abundance of ARG types and subtypes was generated using Hemi1.0. Column diagram depicting the abundance of ARG types were drawn using GraphPad prism 9.0. Petal figure showing specific genes was drawn using Venn (Yang et al., 2022).^3^ Simpson index, Shannon index and Chao1 index (measuring within-sample diversity) were calculated based on ARG relative abundance profiles using the PAST 4.09, and visualization was accomplished using origin (2022) software.

To reduce the false-positive results, False Discovery Rate (FDR) method was applied to adjust *p*-value using JMP 16 (Yang et al., 2022). Network analysis was performed using Gephi (0.10.1) based on significant correlation matrix (*r* > 0.6, *p* < 0.01) (Li et al., 2015; Liu J. W. et al., 2020; Liu et al., 2021; Yang et al., 2023).

## 3 Results

### 3.1 Microbial community annotation of different NHPs

**Figure 1:**
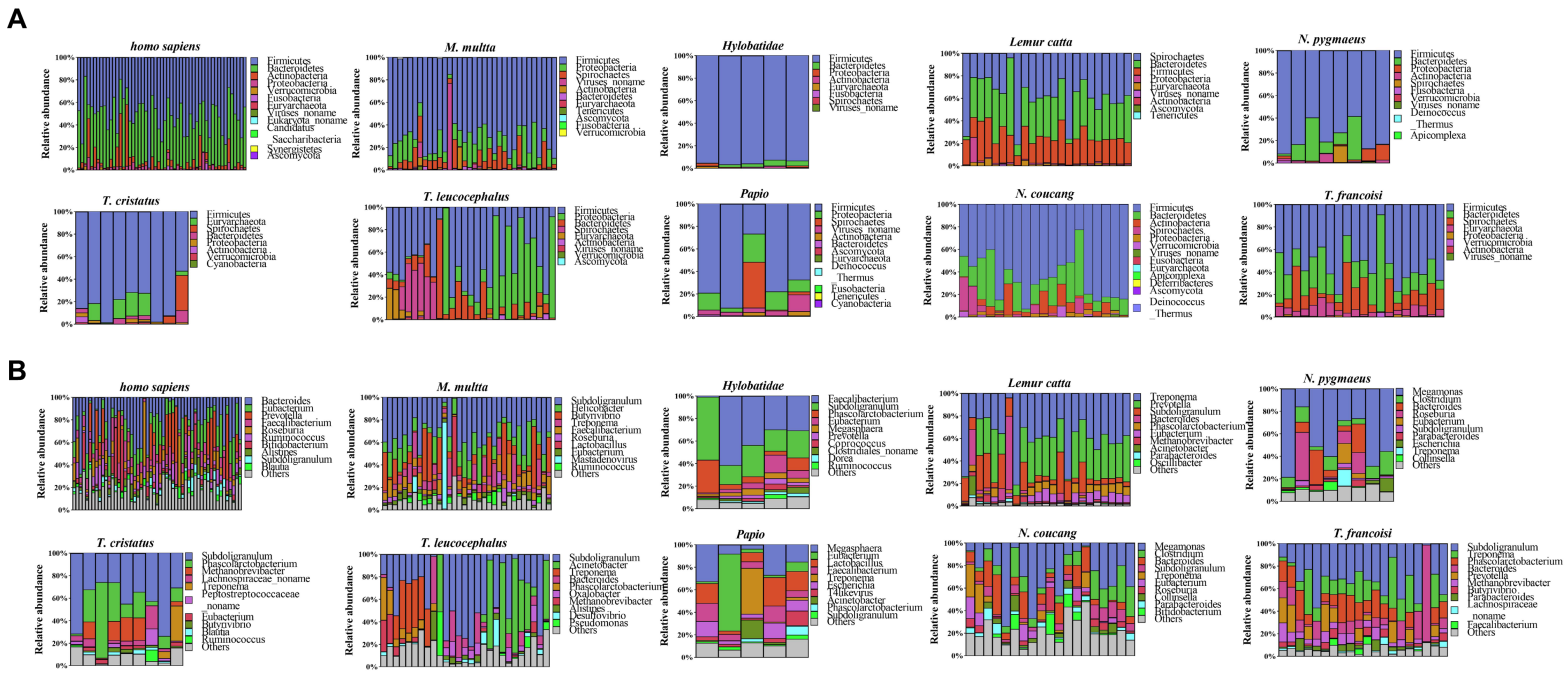
Taxonomic annotation of microbial communities. **(A)** Phylum-level taxonomy annotation. **(B)** Genus-level taxonomy annotation.

In all primate species, bacterial overwhelmingly dominated, contributing a greater degree of phylogenetic diversity compared to archaea, eukaryotes, and viruses (Supplementary Figure S2). At the phylum level, all samples were allocated to 17 distinct phyla, encompassing 14 for NHPs and 12 for humans. Striking similarities emerged between NHPs and humans in terms of their bacterial composition patterns. Notably, within *Homo sapiens*, *T. leucocephalus*, *Hylobatidae*, *Papio*, *M. multta*, *T. francoisi*, *N. pygmaeus*, *T. cristatus*, and *N. coucang*, the predominant phylum was Firmicutes (51, 47, 95, 69, 71, 53, 77, 81 and 65%, respectively), while in *Lemur catta*, the most abundant phylum was Spirochaetes (58.0%). Broadly, Firmicutes, Bacteroidetes, Euryarchaeota, and Proteobacteria constituted the four prevailing phyla across all species, cumulatively accounting for approximately 85.83% of the total bacterial community (Figure 1A).

At the genus level, 228 genera were identified across all samples, showcasing diverse representative genera in each species. A substantial proportion of the Subdoligranulum genus were identified in *Presbytis* (*T. leucocephalus, T. francoisi, T. cristatus*) (56.53, 31.03% and 42.36 respectively), while the predominant genus Megamonas was observed in *Nycticebus* (*N. pygmaeus, N. coucang*) (47.93 and 27.14% respectively). Notably, humans exhibited a uniquely enriched genus, Bacteroides (22%), whereas *Papio* harbored the most abundant genus Megasphaera (17%). In *Hylobatidae,* the leading genus was Faecalibacterium (33%), whereas in *Lemur catta,* the genus Treponema was particularly enriched (38%) (Figure 1B). Furthermore, a total of 85 species-specific genera were identified, each characterized by relatively lower abundances. For instance, *Papio* had 20 such genera, *T. leucocephalus* had 19, *Homo sapiens* had 14, *N. coucang* had 12, *Lemur catta* had 9, *N. pygmaeus* had 4, *T. cristatus* had 3, *M. multta* had 2, and both *Hylobatidae* and *T. francoisi* had 1 each. Additionally, 18 genera were detected as shared across all species, including Subdoligranulum, Bacteroides, Phascolarctobacterium and Methanobrevibacter, which were included in the top 50 most abundant genera (Supplementary Figure S3).

### 3.2 Distinct gut bacterial communities across NHPs

**Figure 2:**
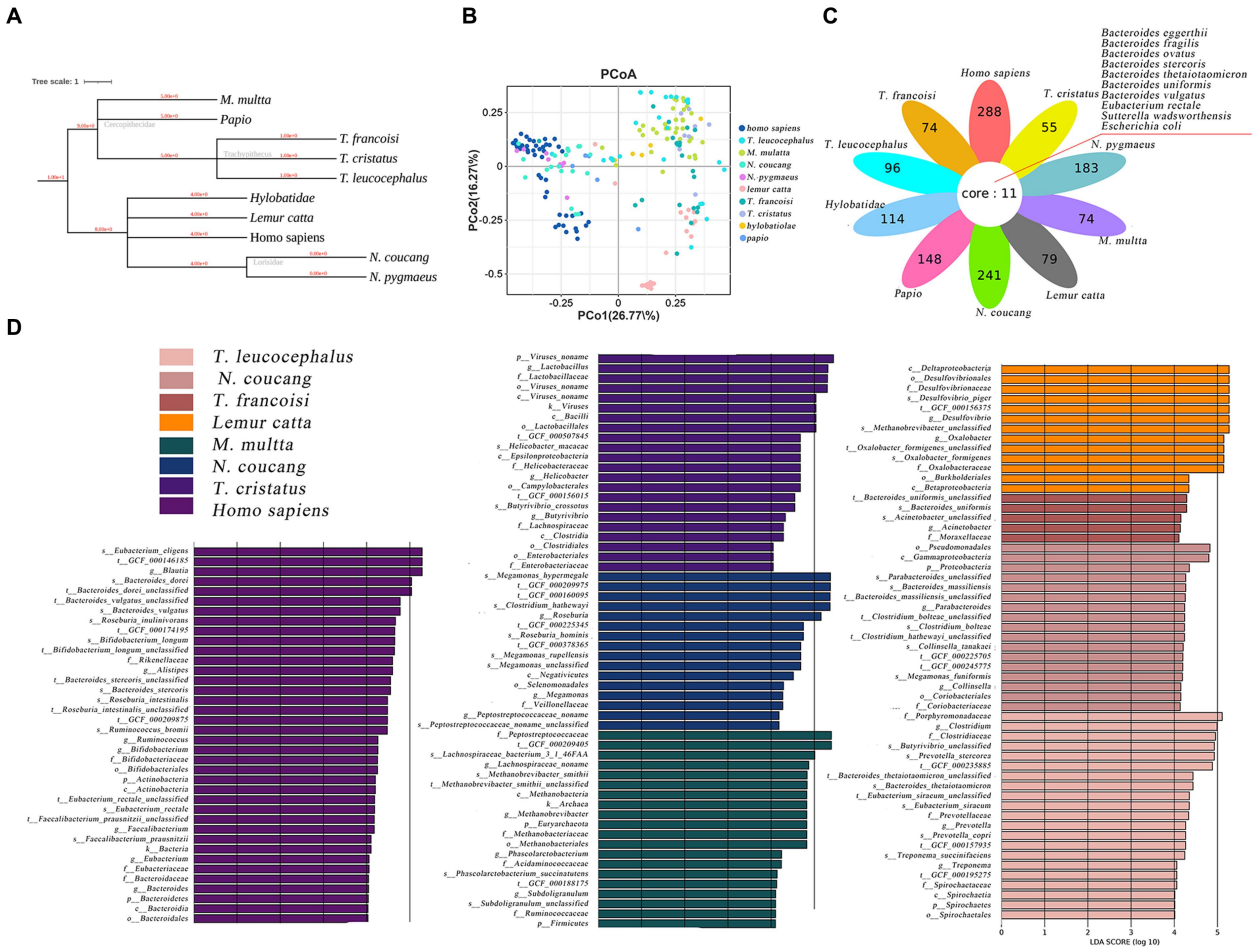
Comparative analysis of gut microbiome among various primate species. **(A)** Hierarchical classification tree of the ten primate species. **(B)** PCoA plot based on Bray-Curtis distance of genus-level relative abundance profile. **(C)** The difference of genus-level relative abundance by LEfSe (*p* < 0.01 and LDA > 4). **(D)** Petal diagram showed shared opportunistic pathogens among the ten species.

The phylogenetic relationships among the ten primate species are depicted in Figure 2A. Utilizing a genus-level relative abundance profile, a PCoA plot elucidated that axis 1 (PCoA1) accounted for 26.77% of the variance, while axis 2 (PCoA2) accounted for 16.27%. *Homo sapiens, N. coucang* and *N. pygmaeus* samples exhibited clustering tendencies, distinct from the remaining samples (Figure 2B). Intriguingly, species sharing genetic host backgrounds demonstrated resemblant gut microbial structures, diverging from prior findings (Cao et al., 2020). Employing LEfSe, we compared the relative abundance of gut bacteria between humans and seven other NHPs species (Supplementary Figure S4). Specifically, 2 phyla (Actinobacteria and Bacteroidetes), 2 classes (Actinobacteria and Bacteroidia), 2 orders (Bacteroidales and Bifidobacteriales), 4 families (Bacteroidaceae, Bifidobacteriaceae, Eubacteriaceae and Rikenellaceae), 7 genera (Alistipes, Bacteroides, Bifidobacterium, Blautia, Eubacterium, Faecalibacterium and Ruminococcus), and 10 species (Ruminococcus_bromii, Roseburia_inulinivorans, Roseburia_intestinalis, Faecalibacterium_prausnitzii, Eubacterium_rectale, Eubacterium_eligens, Bifidobacterium_longum, Bacteroides_vulgatus, Bacteroides_stercoris) exhibited significantly higher abundance in *homo sapiens* (*p* < 0.01 and linear discriminant analysis (LDA) > 4) (Figure 2D).

In total, 107 opportunistic pathogens were detected across all species through comparison with a previously published pathogen list (Li et al., 2015). Among these, 10 pathogens were shared across all ten primates (Figure 2C). *Escherichia coli* and *Bacteroides eggerthii* prevailed most frequently, with *Homo sapiens* (59), *N. coucang* (55) and *N. pygmaeus* (45) hosting the broadest spectrum of pathogens. Clinically pertinent pathogens were identified in NHPs, including *Clostridium perfringens*, *Klebsiella pneumoniae*, and *Clostridium difficile*, which were also found in the migratory birds.

### 3.3 The ARGs and resistant phenotype among NHPs

**Figure 3:**
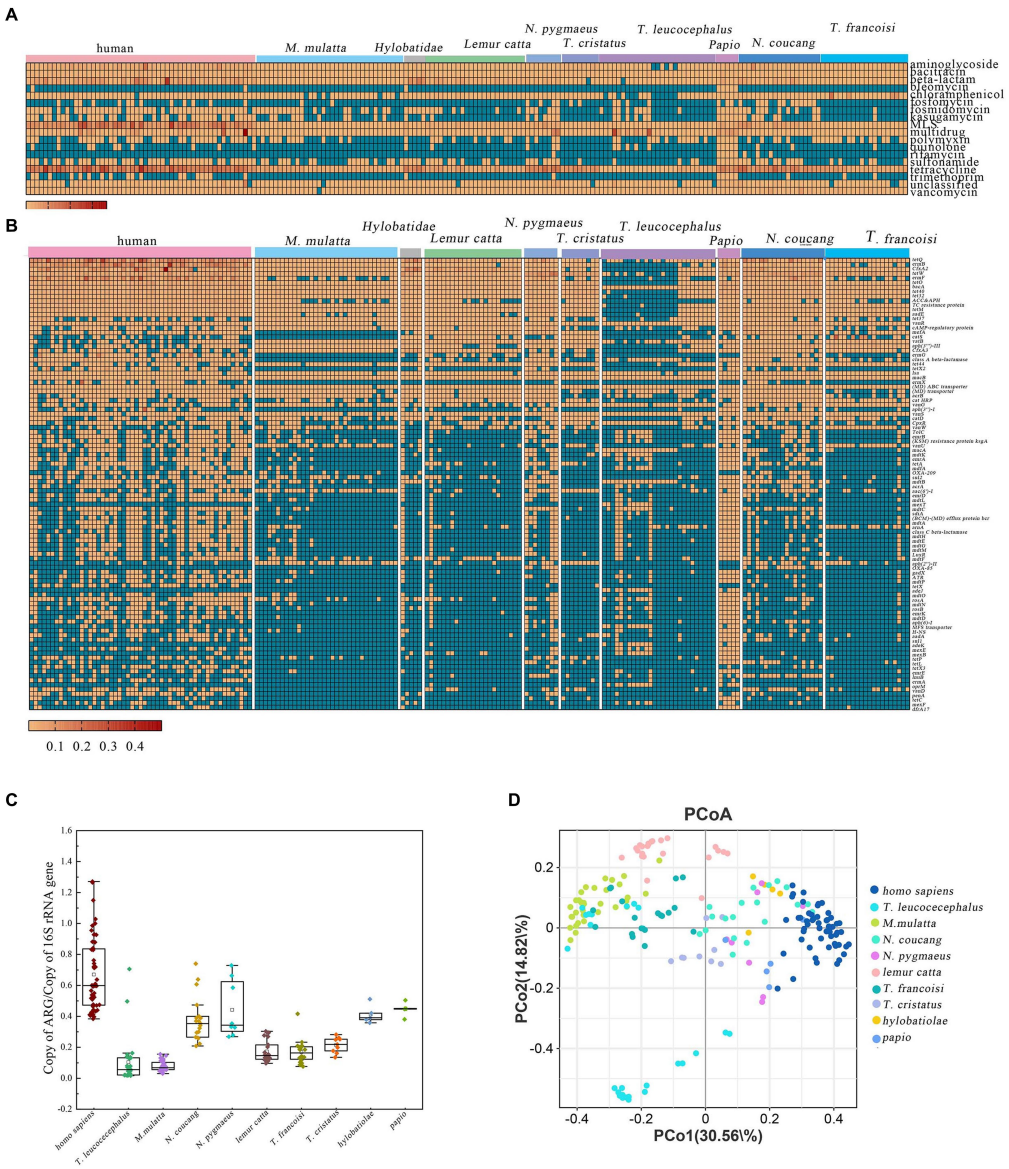
ARGs profiles in different primate species. **(A)** Relative abundance of major ARG types. **(B)** Abundance of the top 100 predominant ARG subtypes in the 203 primate fecal samples. **(C)** Comparative analysis of total ARG abundance across different primate species. **(D)** PCoA based on Bray–Curtis distance of ARG relative abundances.

A total of 18 distinct ARG types were identified across various primate populations. Tetracycline resistance genes comprised the largest proportion at 40% of all detected ARGs, followed by macrolide-lincosamide-streptogramin (MLS) resistance genes (21%), beta-lactam resistance genes (11%), and multidrug resistance genes (8%) (Supplementary Figures S5, S6). The ARGs for Tetracycline, bacitracin, MLS, multidrug, and vancomycin were consistently detected in all samples (Figure 3A). The total abundance of ARGs in the different species exhibited the following ranges: *Homo sapiens* (0.384–1.597), *T. leucocephalus* (9.587 × 10^−6^–4.480 × 10^−1^), *M. multta* (1.023 × 10^−5^–1.040 × 10^−1^), *N. coucang* (1.438 × 10^−5^–3.24 × 10^−1^), *N. pygmaeus* (1.007 × 10^−5^–2.890 × 10^−1^), *Lemur catta* (6.671 × 10^−6^–1.250 × 10^−1^), *T. francoisi* (1.004 × 10^−5^–2.670 × 10^−1^), *T. cristatus* (1.057 × 10^−5^–1.880 × 10^−1^), *Hylobatidae* (2.018 × 10^−5^–2.410 × 10^−1^), and *Papio* (4.513 × 10^−5^–2.20 × 10^−1^) (Figure 3C).

More specifically, within *M. multta*, the dominant resistance genes were tetracycline, MLS, vancomycin, and aminoglycoside, accounting for 65.71, 9.00, 5.23, and 4.61%, respectively, (Supplementary Figure S7A). For *T. leucocephalus*, the foremost resistance genes were multidrug, tetracycline, MLS, and bacitracin, constituting 46.47, 16.26, 9.96, and 8.05%, respectively (Supplementary Figure S6B). In captive *N. coucang* and *N. pygmaeus*, 16 ARGs were observed, with prominent types being tetracycline (49.13, 47.39%), MLS (18.07, 13.96%), and beta-lactam (9.07, 7.02%) (Supplementary Figures 7C,D). Tetracycline, aminoglycoside, MLS, and vancomycin accounted for 42.20, 12.01, 6.69, and 10.82%, respectively, in *T. francoisi,* and 62.90, 12.15, 6.95, and 5.03%, respectively, in *T. cristatus* (Supplementary Figures S6G,H). Among *Hylobatidae* and *Papio*, which exhibited 18 ARGs, tetracycline (46.19%), beta-lactam (32.02%), MLS (5.98%), and bacitracin (5.03%) were predominant in *Hylobatidae* (Supplementary Figure S6F). In *Papio,* tetracycline (39.77%), multidrug (20.41%), MLS (11.95%), and beta-lactam (5.92%) dominated (Supplementary Figures S6I,J). For *Lemur catta*, 18 ARGs were evident, with tetracycline (36.51%), beta-lactam (24.3%), MLS (18.55%), and chloramphenicol (6.64%) as primary resistance genes (Supplementary Figure S7E). Notably, all ARGs annotated in human were also present in the NHPs (Supplementary Figure S7K).

A total of 580 ARGs were identified across all samples, with relative abundances spanning from 3.01×10^−6^ (TEM-201) to 10.07 (t*et*Q). The heatmap showcased the top 100 most abundant ARGs, comprising 34 classified as multidrug resistance, 16 as tetracycline, 11 as MLS, and 8 as aminoglycoside (Figure 2B). *van*R and *mac*B were universally shared across all samples, indicating their ubiquitous presence among primates. Tetracycline-related ARGs, including *tet*35, *tet*36, *tet*37, *tet*39, *tet*40, *tet*44, *tet*A, *tet*L, *tet*M, *tet*O, *tet*P, *tet*Q, tetracycline_resistance_protein, *tet*W, *tet*X1, and *tet*X2, exhibited high abundances in captive primates. Furthermore, a total 93 ARGs were shared among the ten primate species, with *Papio* harboring the most diverse ARGs (355) (Supplementary Figure S8).

### 3.4 Comparative analysis of resistome between NPHs and humans

**Figure 4:**
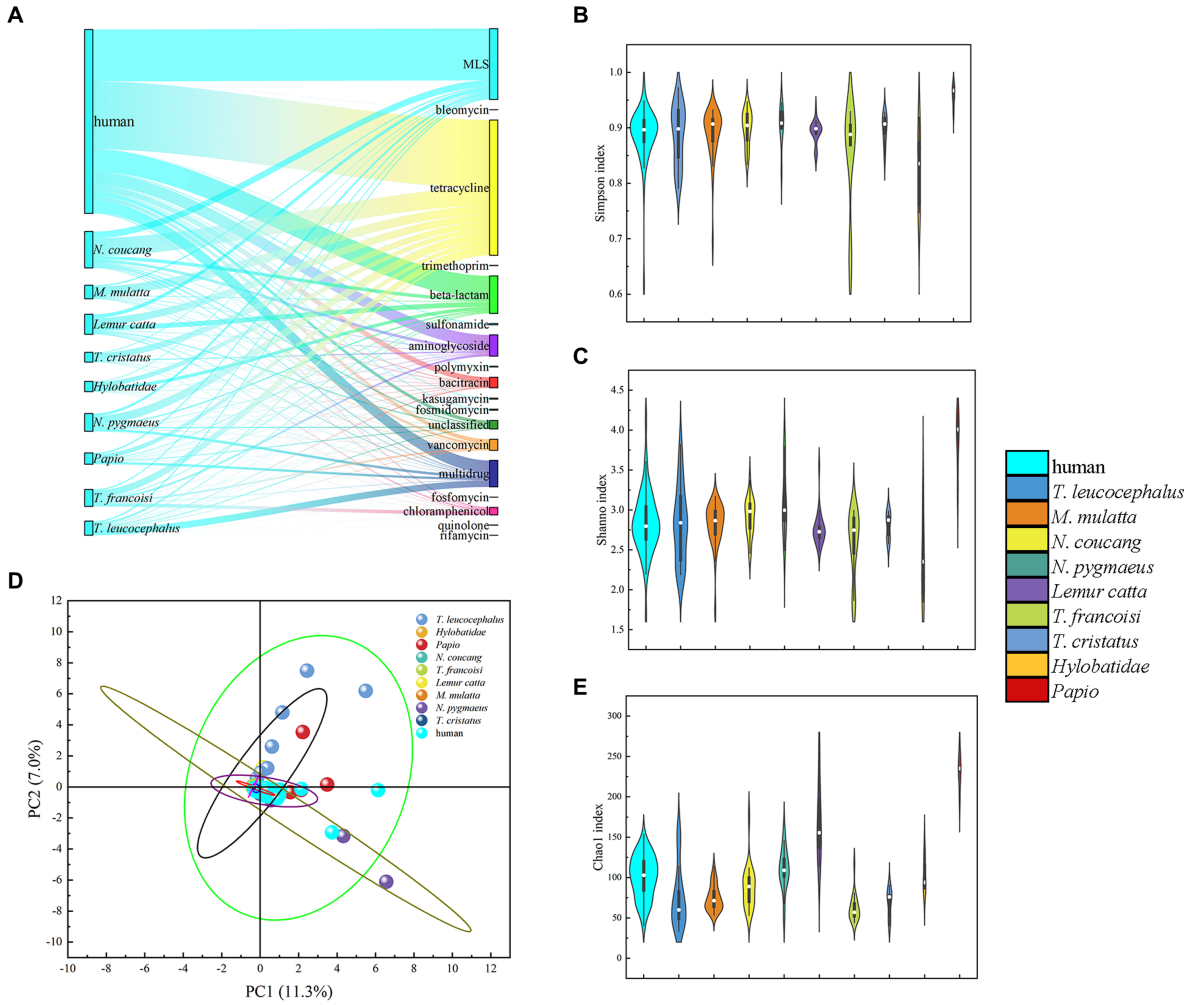
Comparative analysis of gut antibiotic resistome among various primate species. **(A)** Comparison of ARG types and proportion in the nine NHPs and human. **(B)** Simpson index Comparison of ARGs in NHPs and human. **(C)** Shannon indexes Comparison of ARGs in NHPs and human. **(D)** PCA plot illustrating ARG types among NHPs and human based on relative abundance. **(E)** Chao1 index comparison of ARGs in NHPs and human.

Previous studies have underscored the close resemblance between the gut microbiomes of NHPs and humans, suggesting the potential utility of NHPs in biomedical research as model (Campbell et al., 2020; Yang et al., 2022). Building on this premise, we conducted a comparison of antibiotic resistome composition and diversity indices between nine NHPs and humans adhering to a non-westernized diet. Our analysis revealed the existence of 16 ARGs shared between both humans and NHPs. Moreover, rifamycin resistance genes were exclusively identified in NHPs, albeit at a limited frequency. Among these genes, tetracycline stood out as the most prevalent and dominant ARGs in metagenomic resistome profiles of both humans and NHPs, followed by MLS, beta-lactam, and multidrug (Figure 4A).

Diversity index analysis demonstrated that human exhibited relatively higher Simpson, Shannon, and Chao1 indices, compared to NHPs. Notably, captive *Papio* displayed the highest diversity indexes. PCA plots based on ARGs relative abundance indicated that nearly all NHPs samples (particularly those from *T. francoisi*, *T. cristatus*, *N. pygmaeus*, and *M. mulatta*) were closely situated to human samples (Figure 4D; Supplementary Figures S9B–D,F). However, some *T. leucocephalus* and *Lemur catta* samples diverged from human samples in the PCA plot (Supplementary Figures S10A,E). A considerable abundance of ARGs was identified in samples from the Wuzhou Langur Breeding and Research Center and Nanning Zoon’s *T. francoisi* (Supplementary Figure S9). consequently, it can be inferred that lifestyles and geography exert an influence on similarities and abundance of ARGs among the NHPs.

Thus, NHPs resistomes represent a potentially crucial reservoir for studying antibiotic resistance within human gut microbiota. This warrants attention as captive NHPs frequently interact with humans, raising concerns for potential public health implications. Furthermore, it is noteworthy that both wild *T. leucocephalus* and *M. mulatta* harbor numerous ARGs that align with those found in human beings.

### 3.5 Co-occurrence network analysis of ARGs in NHPs

**Figure 5:**
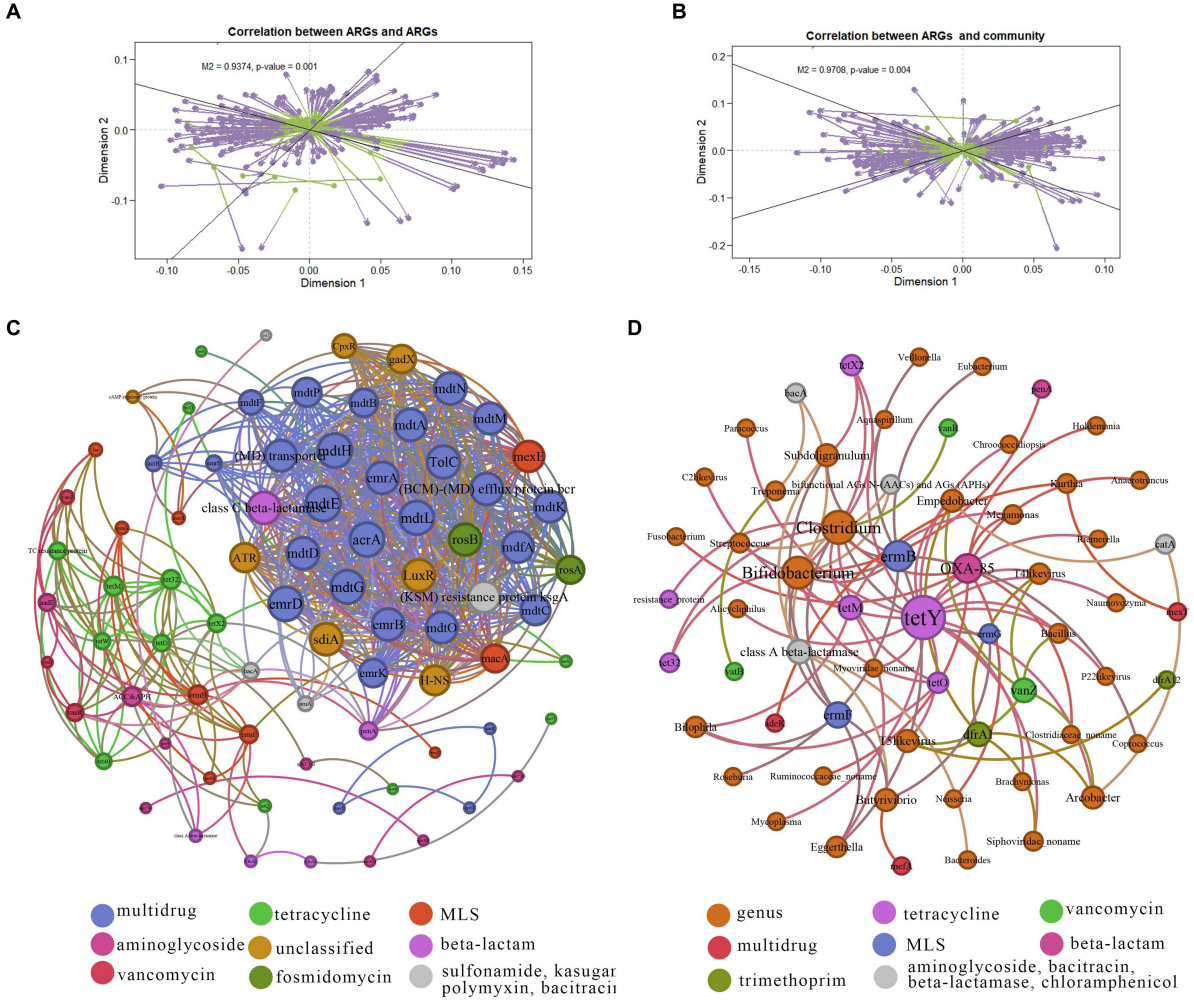
Co-occurrence networks of ARGs and microbiota taxa. **(A)** Procrustes analysis results showing correlations between ARGs. **(B)** Procrustes analysis results demonstrating correlations between ARG composition and microbial taxonomy. **(C)** Network analysis illustrating co-occurrence patterns among ARG subtypes. Node size indicates connection strength (degree) with strong (Spearman’s correlation coefficient *ρ* > 0.6) and significant (*p* value <0.01) correlations. **(D)** Network analysis illustrating co-occurrence patterns between ARG subtypes and microbial taxa. Node size indicates connection strength (degree) with strong (Spearman’s correlation coefficient *ρ* > 0.6) and significant (*p* value <0.01) correlations.

Procrustes analysis was employed to elucidate correlations between ARG subtypes and bacteria taxa at the genus level. Deviation square *M*^2^ yielded a value of 0.9374, with a corresponding *p* value 0.001 for ARGs and ARGs (Figure 5A). Additionally, the deviation square *M*^2^ for ARGs and microbiota amounted to 0.9708, with *p* value of 0.001 (Figure 5B).

To unravel the co-occurrence patterns of ARGs within the ten NHPs, a co-occurrence network was constructed based on robust and statistically significant correlations among different NPHs found in individuals (|ρ| > 0.6, *p* < 0.01). The co-occurrence network diagrams for ARGs contained 12 antibiotic types encompassing 79 resistance genes (Figure 5C). Positive correlations emerged among similar ARGs indicative of the shared selective pressure imposed by specific antibiotics. For example, tetracycline ARGs *tet*O, *tet*M, *tet*32, *tet*X2, *tet*X and *tet*X3 exhibited such correlations. Similar patterns were observed for tetracycline-related ARGs *van*R and *van*S. Only one negative correlation was observed between different ARGs, such as *bac*A (bacitracin ARG) and *vat*B (MLS ARG). This could be attributed to competition among host bacteria or inhibition-related dynamics (Yan et al., 2022).

Illustrated in Figure 5D, the network comprised 61 nodes and 84 edges (with a modularity index of 0.602), including Bifidobacterium, Clostridium, Subdoligranulum, and Ruminococcaceae_noname. Furthermore, 38 species levels were linked to 10 resistance gene types, encompassing tetracycline, vancomycin, aminoglycoside, bacitracin, multidrug, MLS, beta-lactamase, beta-lactam, trimethoprim and chloramphenicol. Notably, Treponema exhibited a positive correlation with *vat*B (MLS ARG), indicating that its potential role as a host for ARGs. Concurrently, certain species displayed negative correlation with ARGs, exemplified by the Spirochaetes phylum, Treponema, and *bac*A (bacitracin ARG). Thus, intricate interaction between various ARGs and microbes were evident, underscoring the intimate connection between NHP gut microbes and ARGs. Besides, based on these results we can tract the hosts for specific ARG subtypes (Li et al., 2023).

## 4 Discussion

NHPs, situated proximately to humans within the animal kingdom, share a substantial degree of genetic and physiological resemblance (Li et al., 2018; Yan et al., 2022). Exploring and contrasting the gut microbiota and antibiotic resistome within captive and wild primate populations can yield valuable insights into human interaction and captivity on NHPs (Campbell et al., 2020). Consequently, a comprehensive understanding of the gut microbiota and ARGs across diverse NHPs and their distinctions from humans is of notable import. Nevertheless, the existing research in this domain remains limited. In this study, we observed a greater similarity in gut microbial composition particularly between captive NHPs (*N. coucang, N. pygmaeus*) and humans. Our investigation revealed 107 opportunistic pathogens and 580 distinct ARGs, unveiling active correlation between the ARGs to ARGs, as well as ARGs and microbiota at genus level, across ten primate species.

We established that Firmicutes, Bacteroidetes, Euryarchaeota and Proteobacteria emerged as the dominant phyla in NHPs. Notably, the gut microbiome clustering exhibited species affiliations within specific geographic region. Human gut microbial composition exhibited a closer affinity to that of *N. coucang* and *N. pygmaeus* compared to other NHPs. As such, we infer that NHPs captivity may foster similarities in gut microbiomes akin to humans. Previous study reconstructed over 1,000 previously uncharacterized microbial species from 6 available NHP metagenomic cohorts, almost 90% of which associated with NHPs, suggesting that captivity possibly humanizes the primate intestinal microbiome (Manara et al., 2019). However, Li et al. (2018) identified in total 1.9 million non-redundant bacterial genes from twenty cynomolgus macaques of which only 39.49% is present in the human. We realize that discernible differences persist in the gut microbiota of captive NHPs and modern humans following diverse diets (Yang et al., 2022). This observation aligns with our identification of 10 species (Ruminococcus_bromii, Roseburia_inulinivorans, Roseburia_intestinalis, Faecalibacterium_prausnitzii, Eubacterium_rectale, Eubacterium_eligens, Bifidobacterium_longum, Bacteroides_vulgatus, Bacteroides_stercoris) that exhibited significantly greater abundance within the human gut, as determined through LEfSe analysis.

Furthermore, our investigation unveiled a notable trend of tetracycline and beta-lactam resistance among captive primates. Remarkably, tetracycline resistance was also discerned in *T. leucocephalus*, albert at a proportion lower than that in *M. mulatta*. Tetracycline and beta-lactam, recognized as broad-spectrum antibiotics, find primary use as first-line treatments for non-communicable diseases resulting from bacterial infections (Fair and Tor, 2014; Hu et al., 2021). Their antibacterial spectrum encompasses Gram-positive and Gram-negative bacteria, chlamydia, mycoplasma, rickettsia, spirochetes, mycobacteria, and some protozoa. Consequently, tetracycline and beta-lactam are extensively employed in veterinary clinics for dogs, cats, cattle, sheep, pigs, turkey, and chickens. This widespread application potentially underpins the elevated prevalence of tetracycline and beta-lactam resistance among captive NHPs. Among the 17 identified tetracycline-related ARGs, *tet*X stands out as an enzyme modification gene, capable of inactivating antibiotic enzymes through enzymatic hydrolysis. Notable, *tet*Q, *tet*W, *tet*O, *tet*32, and *tet*44 genes, which are also prevalent resistance genes in pigs and cattle, manifest substantial abundance (Lim et al., 2020). For instance, *tet*32, initially identified in *Clostridium difficile*-associated human colonic anaerobe K10, finds wide distribution in bovine rumen and pig feces (Cao et al., 2020). The identification of numerous tetracycline-related ARGs underscores the necessity for heightened resistance surveillance and judicious antibiotic usage. Strategies such as alternative antibiotic selection or modifications in the living and dietary conditions of NHPs warrant consideration as control measures.

Co-occurrence network analysis offers a high-resolution depiction of correlations between ARGs and microbiota; however, it does not elucidate definitive relationships. Nonrandom symbiosis models can be used to elucidate potential hosts of ARGs within animal feces and environment samples. Essentially, this nonrandom symbiosis pattern suggests that specific microbiota may harbor distinct ARGs. In our study, we postulated that when the abundance trends of ARGs align significantly with coexisting microbiota across different species, a nonrandom co-occurrence pattern potentially exists between ARGs and microbiota. This led us to infer that Clostridium (with 11 ARG subtypes), Bifidobacterium (with 10 ARG subtypes), and T4likevirus (with 7 ARG subtypes) might emerge as primary potential hosts for tetracycline and MLS. Given that these ARG-carrying bacteria may pose substantial risks to animal health, and potentially transmit to humans, it becomes imperative to implement stringent control measures to mitigate these concerns and curtail the risk of therapeutic failure. Several potential ARGs hosts were confirmed through experimental validation, such as *mdt*O in Escherichia, underscoring the value of co-occurrence network analysis as a robust analytical tool capable of unearthing fresh insights into ARGs and their potential hosts within intricate environmental systems. Nevertheless, these potential symbiosis relationships necessitate further exploration through alternate methods, such as metagenomic assembly and microbiological cultivation.

Compared to human, we observed a resemblance in human gut ARGs to those present in captive NHPs, with numerous overlapping ARG detected in both populations. An investigation involving chimpanzees, gorillas, and humans with different lifestyles reported a segregation of ARGs in wild chimpanzees and gorillas, while captive individuals exhibited a clustering closer to humans (Campbell et al., 2020). Our result further revealed an elevated ARGs abundance in captive primates compared to their wild counterparts. Concurrently, primate species within the same geographic region, such as *N. coucang* and *N. pygmaeus*, exhibited similarities in their ARG profiles, whereas significant differences in ARGs abundance were evident in *T. francoisi* across distinct regions. Remarkably, the detected ARG in humans were also observed in NHPs, underscoring the notable impact of lifestyle and geographic location on gut microbiota-associated ARGs. Moreover, the dispersion of ARGs in *T. leucocephalus,* with certain samples approximating the human pattern, potentially signifies interactions with humans, possibly through contact with agricultural products. In totality, our findings suggest that lifestyle and geographical location exert substantial influence on NHPs ARGs dynamics.

Recognizing areas warranting refinement, we acknowledge the necessity for multivariate analysis to unveil the distinct contributions of lifestyle and geographic factors to the variability of ARG profiles. While our study presents a pioneering exploration of the ARG composition in *T. leucocephalus* and its comparison to humans, the inclusion of only one captive *T. leucocephalus* precludes a comprehensive analysis of the specific impact of the karst environment on ARGs. In our analyses, spearman correlation elucidated associations between ARG and genus-level microbiota. However, it is crucial to note that numerous ARGs can transfer among bacteria through self-proliferation and horizontal gene transfer in real-world settings. Therefore, certain genes, particularly those widely distributed across bacterial species, might not be solely discerned through correlation analysis (Liu et al., 2019). Consequently, further investigations are warranted to deepen our insights in subsequent studies. Regardless, our study unveils the gut microbiota and resistome of different primates by metagenomic sequencing, providing essential evidence for NHPs and humans to combat the threat of antibiotic resistance.

## 5 Conclusion

Through comprehensive metagenomic analysis of ten primates sequencing datasets, we have established an extensive and diverse repository of primate gut microbiota and ARGs. Our study underscores the potential influence of factors like captivity, geographic location, and species on the composition of gut microbiota and ARGs in NHPs. Notably, captive NHPs exhibited elevated relative abundance of ARGs compared to their wild counterparts. Moreover, our co-occurrence network analysis revealed discernible correlation between ARGs and distinct microbiota components. This research enriches our comprehension of the parallels and distinctions in gut microbiota and ARGs profiles between NHPs and humans, offering a pivotal reference for future investigation in this domain.

## Data availability statement

The datasets presented in this study can be found in online repositories. The names of the repository/repositories and accession number(s) can be found in the article/Supplementary material.

## Author contributions

HH: Data curation, Methodology, Software, Writing – original draft, Writing – review & editing. XP: Conceptualization, Formal analysis, Software, Writing – review & editing. TQ: Investigation, Project administration, Resources, Writing – review & editing. PC: Data curation, Resources, Visualization, Writing – review & editing. SL: Resources, Visualization, Writing – review & editing. AW: Resources, Visualization, Writing – review & editing. MH: Resources, Visualization, Writing – review & editing. HQ: Resources, Visualization, Writing – review & editing. YH: Funding acquisition, Project administration, Supervision, Validation, Writing – original draft.

## Footnotes

^1^ https://www.ebi.ac.uk/ena/browser/view/

^2^ http://smile.hku.hk/SARGs/

^3^ http://www.ehbio.com/test/venn/#/

## References

[ref1] Allen H. K. Donato J. Wang H. H. Cloud-Hansen K. A. Davies J. Handelsman J. (2010). Call of the wild: antibiotic resistance genes in natural environments. Nat. Rev. Microbiol. 8, 251–259. doi: 10.1038/nrmicro2312, PMID: 20190823

[ref2] Bolan G. A. Sparling P. F. Wasserheit J. N. (2012). The emerging threat of untreatable gonococcal infection. N. Engl. J. Med. 366, 485–487. doi: 10.1056/NEJMp1112456, PMID: 22316442

[ref3] Brinkac L. Voorhies A. Gomez A. Nelson K. E. (2017). The threat of antimicrobial resistance on the human microbiome. Microb. Ecol. 74, 1001–1008. doi: 10.1007/s00248-017-0985-z, PMID: 28492988 PMC5654679

[ref4] Campbell T. P. Sun X. Patel V. H. Sanz C. Morgan D. Dantas G. (2020). The microbiome and resistome of chimpanzees, gorillas, and humans across host lifestyle and geography. ISME J. 14, 1584–1599. doi: 10.1038/s41396-020-0634-2 32203121 PMC7242348

[ref5] Cao J. Hu Y. Liu F. Wang Y. Bi Y. Lv N.. (2020). Metagenomic analysis reveals the microbiome and resistome in migratory birds. Microbiome 8:26. doi: 10.1186/s40168-019-0781-8, PMID: 32122398 PMC7053137

[ref6] Chi T. Zhang A. Zhang X. Li A. D. Zhang H. Zhao Z. (2020). Characteristics of the antibiotic resistance genes in the soil of medical waste disposal sites. Sci. Total Environ. 730:139042. doi: 10.1016/j.scitotenv.2020.139042, PMID: 32402966

[ref7] Fair R. J. Tor Y. (2014). Antibiotics and bacterial resistance in the 21st century. Perspect Med Chem 6, 25–64. doi: 10.4137/PMC.S14459 PMC4159373 25232278

[ref8] Fan Y. Pedersen O. (2021). Gut microbiota in human metabolic health and disease. Nat. Rev. Microbiol. 19, 55–71. doi: 10.1038/s41579-020-0433-9 32887946

[ref9] Feng J. Li B. Jiang X. Yang Y. Wells G. F. Zhang T.. (2018). Antibiotic resistome in a large-scale healthy human gut microbiota deciphered by metagenomic and network analyses. Environ. Microbiol. 20, 355–368. doi: 10.1111/1462-2920.14009, PMID: 29194931

[ref10] Gacesa R. Kurilshikov A. Vich Vila A. Sinha T. Klaassen M. A. Y. Bolte L. A.. (2022). Environmental factors shaping the gut microbiome in a Dutch population. Nature 604, 732–739. doi: 10.1038/s41586-022-04567-7, PMID: 35418674

[ref11] Gil-Gil T. Ochoa-Sánchez L. E. Baquero F. Martínez J. L. (2021). Antibiotic resistance: time of synthesis in a post-genomic age. Comput. Struct. Biotechnol. J. 19, 3110–3124. doi: 10.1016/j.csbj.2021.05.034, PMID: 34141134 PMC8181582

[ref12] Guo W. Mishra S. Wang C. Zhang H. Ning R. Kong F.. (2019). Comparative study of gut microbiota in wild and captive Giant pandas (Ailuropoda melanoleuca). Genes (Basel). 10:827. doi: 10.3390/genes10100827, PMID: 31635158 PMC6826394

[ref13] He L. Y. He L. K. Liu Y. S. Zhang M. Zhao J. L. Zhang Q. Q.. (2019). Microbial diversity and antibiotic resistome in swine farm environments. Sci. Total Environ. 685, 197–207. doi: 10.1016/j.scitotenv.2019.05.369, PMID: 31174117

[ref14] Hu T. Dai Q. Chen H. Zhang Z. Dai Q. Gu X.. (2021). Geographic pattern of antibiotic resistance genes in the metagenomes of the giant panda. Microb. Biotechnol. 14, 186–197. doi: 10.1111/1751-7915.13655, PMID: 32812361 PMC7888472

[ref15] Huang C. M. Li Y. B. (2005). How does the white-headed langur (Trachypithecus leucocephalus) adapt locomotor behavior to its unique limestone hill habitat? Primates 46, 261–267. doi: 10.1007/s10329-005-0130-3, PMID: 15915324

[ref16] Li Y. B. Huang X. H. Huang Z. H. (2020). Behavioral adjustments and support use of Francois' langur in limestone habitat in Fusui, China: implications for behavioral thermoregulation. Ecol. Evol. 10, 4956–4967. doi: 10.1002/ece3.6249, PMID: 32551073 PMC7297789

[ref17] Li B. Ju F. Cai L. Zhang T. (2015). Profile and fate of bacterial pathogens in sewage treatment plants revealed by high-throughput metagenomic approach. Environ. Sci. Technol. 49, 10492–10502. doi: 10.1021/acs.est.5b02345, PMID: 26252189

[ref18] Li X. P. Liang S. Xia Z. Qu J. Liu H. Liu C.. (2018). Establishment of a gut microbiome gene catalog and comparison with the human, pig, and mouse gut microbiomes. Gigascience 7:giy100. doi: 10.1093/gigascience/giy100, PMID: 30137359 PMC6137240

[ref19] Li B. Yang Y. Ma L. Ju F. Guo F. Tiedje J. M.. (2015). Metagenomic and network analysis reveal wide distribution and co-occurrence of environmental antibiotic resistance genes. ISME J. 9, 2490–2502. doi: 10.1038/ismej.2015.59, PMID: 25918831 PMC4611512

[ref20] Li Z. H. Yuan D. Kou Y. Li X. Du C. (2023). Metagenome sequencing to unveil the occurrence and distribution of antibiotic resistome and in a wastewater treatment plant. Environ. Technol., 1–10. doi: 10.1080/09593330.2022.2158758 36812908

[ref21] Lim S. K. Kim D. Moon D. C. Cho Y. Rho M. (2020). Antibiotic resistomes discovered in the gut microbiomes of Korean swine and cattle. Gigascience 9:giaa043. doi: 10.1093/gigascience/giaa043, PMID: 32369165 PMC7317084

[ref22] Liu Y. X. Qin Y. Chen T. Lu M. Qian X. Guo X.. (2021). A practical guide to amplicon and metagenomic analysis of microbiome data. Protein Cell 12, 315–330. doi: 10.1007/s13238-020-00724-8, PMID: 32394199 PMC8106563

[ref23] Liu J. X. Taft D. H. Maldonado-Gomez M. X. Johnson D. Treiber M. L. Lemay D. G.. (2019). The fecal resistome of dairy cattle is associated with diet during nursing. Nat Communicat 10:4406. doi: 10.1038/s41467-019-12111-x PMC6765000 31562300

[ref24] Liu Y. Y. Wang Y. Walsh T. R. Yi L. X. Zhang R. Spencer J.. (2016). Emergence of plasmid-mediated colistin resistance mechanism MCR-1 in animals and human beings in China: a microbiological and molecular biological study. Lancet Infect. Dis. 16, 161–168. doi: 10.1016/S1473-3099(15)00424-7, PMID: 26603172

[ref25] Liu Z. J. Zhang L. Yan Z. Ren Z. Han F. Tan X.. (2020). Genomic mechanisms of physiological and morphological adaptations of limestone langurs to karst habitats. Mol. Biol. Evol. 37, 952–968. doi: 10.1093/molbev/msz301, PMID: 31846031

[ref26] Liu J. W. Zhu S. Liu X. Yao P. Ge T. Zhang X. H. (2020). Spatiotemporal dynamics of the archaeal community in coastal sediments: assembly process and co-occurrence relationship. ISME J. 14, 1463–1478. doi: 10.1038/s41396-020-0621-7, PMID: 32132664 PMC7242467

[ref27] Manara S. Asnicar F. Beghini F. Bazzani D. Cumbo F. Zolfo M.. (2019). Microbial genomes from non-human primate gut metagenomes expand the primate-associated bacterial tree of life with over 1000 novel species. Genome Biol. 20:299. doi: 10.1186/s13059-019-1923-9, PMID: 31883524 PMC6935492

[ref28] Mustafa G. R. Li C. Zhao S. Jin L. He X. Shabbir M. Z.. (2021). Metagenomic analysis revealed a wide distribution of antibiotic resistance genes and biosynthesis of antibiotics in the gut of giant pandas. BMC Microbiol. 21:15. doi: 10.1186/s12866-020-02078-x, PMID: 33413128 PMC7792088

[ref29] Orkin J. D. Campos F. A. Myers M. S. Cheves Hernandez S. E. Guadamuz A. Melin A. D. (2019). Seasonality of the gut microbiota of free-ranging white-faced capuchins in a tropical dry forest. ISME J. 13, 183–196. doi: 10.1038/s41396-018-0256-0, PMID: 30135468 PMC6298967

[ref30] Qian X. Gunturu S. Guo J. Chai B. Cole J. R. Gu J.. (2021). Metagenomic analysis reveals the shared and distinct features of the soil resistome across tundra, temperate prairie, and tropical ecosystems. Microbiome 9:108. doi: 10.1186/s40168-021-01047-4, PMID: 33990222 PMC8122544

[ref31] Qiao M. Ying G. G. Singer A. C. Zhu Y. G. (2018). Review of antibiotic resistance in China and its environment. Environ. Int. 110, 160–172. doi: 10.1016/j.envint.2017.10.016, PMID: 29107352

[ref32] Qiu Q. W. Wang J. Yan Y. Roy B. Chen Y. Shang X.. (2020). Metagenomic analysis reveals the distribution of antibiotic resistance genes in a large-scale population of healthy individuals and patients with varied diseases. Front. Mol. Biosci. 7:18. doi: 10.3389/fmolb.2020.590018, PMID: 33330625 PMC7673455

[ref33] Que T. C. Wang H. Yang W. Wu J. Hou C. Pei S.. (2021). The reference genome and transcriptome of the limestone langur, Trachypithecus leucocephalus, reveal expansion of genes related to alkali tolerance. BMC Biol. 19:67. doi: 10.1186/s12915-021-00998-2, PMID: 33832502 PMC8034193

[ref34] Roca I. Akova M. Baquero F. Carlet J. Cavaleri M. Coenen S.. (2015). The global threat of antimicrobial resistance: science for intervention. New Microbes New Infect 6, 22–29. doi: 10.1016/j.nmni.2015.02.007, PMID: 26029375 PMC4446399

[ref35] Schuchat A. Hilger T. Zell E. Farley M. M. Reingold A. Harrison L.. (2001). Active bacterial core surveillance of the emerging infections program network. Emerg. Infect. Dis. 7, 92–99. doi: 10.3201/eid0701.010114, PMID: 11266299 PMC2631675

[ref36] Stange C. Tiehm A. (2020). Occurrence of antibiotic resistance genes and microbial source tracking markers in the water of a karst spring in Germany. Sci. Total Environ. 742:140529. doi: 10.1016/j.scitotenv.2020.140529, PMID: 32629259

[ref37] Su Z. G. Wen D. Gu A. Z. Zheng Y. Tang Y. Chen L. (2023). Industrial effluents boosted antibiotic resistome risk in coastal environments. Environ. Int. 171:107714. doi: 10.1016/j.envint.2022.107714, PMID: 36571993

[ref38] Sun Y. M. Qiu T. Gao M. Shi M. Zhang H. Wang X. (2019). Inorganic and organic fertilizers application enhanced antibiotic resistome in greenhouse soils growing vegetables. Ecotoxicol. Environ. Saf. 179, 24–30. doi: 10.1016/j.ecoenv.2019.04.039, PMID: 31022652

[ref39] van Boeckel T. P. Brower C. Gilbert M. Grenfell B. T. Levin S. A. Robinson T. P.. (2015). Global trends in antimicrobial use in food animals. Proc. Natl. Acad. Sci. U. S. A. 112, 5649–5654. doi: 10.1073/pnas.1503141112, PMID: 25792457 PMC4426470

[ref40] Wang C. L. Li P. Yan Q. Chen L. Li T. Zhang W.. (2019). Characterization of the pig gut microbiome and antibiotic resistome in industrialized feedlots in China. Msystems 4:e00206–19. doi: 10.1128/mSystems.00206-19, PMID: 31848308 PMC6918024

[ref41] Xiang S. Z. Wang X. Ma W. Liu X. Zhang B. Huang F.. (2020). Response of microbial communities of karst river water to antibiotics and microbial source tracking for antibiotics. Sci. Total Environ. 706:135730. doi: 10.1016/j.scitotenv.2019.135730, PMID: 31791761

[ref42] Xu Y. Xu J. Mao D. Luo Y. (2017). Effect of the selective pressure of sub-lethal level of heavy metals on the fate and distribution of ARGs in the catchment scale. Environ. Pollut. 220, 900–908. doi: 10.1016/j.envpol.2016.10.074, PMID: 27876226

[ref43] Yan Y. Y. Li H. Fayyaz A. Gai Y. (2022). Metagenomic and network analysis revealed wide distribution of antibiotic resistance genes in monkey gut microbiota. Microbiol. Res. 254:126895. doi: 10.1016/j.micres.2021.126895, PMID: 34742104

[ref44] Yang Y. Y. Li Z. Song W. du L. Ye C. Zhao B.. (2019). Metagenomic insights into the abundance and composition of resistance genes in aquatic environments: influence of stratification and geography. Environ. Int. 127, 371–380. doi: 10.1016/j.envint.2019.03.062, PMID: 30954723

[ref45] Yang S. Liu Y. Yang N. Lan Y. Lan W. Feng J.. (2022). The gut microbiome and antibiotic resistome of chronic diarrhea rhesus macaques (Macaca mulatta) and its similarity to the human gut microbiome. Microbiome 10:29. doi: 10.1186/s40168-021-01218-3, PMID: 35139923 PMC8827259

[ref46] Yang J. T. Tong C. Xiao D. Xie L. Zhao R. Huo Z.. (2022). Metagenomic insights into chicken gut antibiotic Resistomes and microbiomes. Microbiology Spectrum 10:e0190721. doi: 10.1128/spectrum.01907-21, PMID: 35230155 PMC9045286

[ref47] Yang F. X. Wang X. Tian X. Zhang Z. Zhang K. Zhang K.. (2023). Cow manure simultaneously reshaped antibiotic and metal resistome in the earthworm gut tract by metagenomic analysis. Sci. Total Environ. 856:159010. doi: 10.1016/j.scitotenv.2022.159010, PMID: 36174681

[ref48] Yildirim S. Yeoman C. J. Sipos M. Torralba M. Wilson B. A. Goldberg T. L.. (2010). Characterization of the fecal microbiome from non-human wild Primates reveals species specific microbial communities. PLoS One 5:e13963. doi: 10.1371/journal.pone.0013963, PMID: 21103066 PMC2980488

[ref49] Yin X. L. Jiang X. T. Chai B. Li L. Yang Y. Cole J. R.. (2018). ARGs-OAP v2.0 with an expanded SARG database and hidden Markov models for enhancement characterization and quantification of antibiotic resistance genes in environmental metagenomes. Bioinformatics 34, 2263–2270. doi: 10.1093/bioinformatics/bty053, PMID: 29408954

[ref50] Zhang Q. Q. Ying G. G. Pan C. G. Liu Y. S. Zhao J. L. (2015). Comprehensive evaluation of antibiotics emission and fate in the river basins of China: source analysis, multimedia modeling, and linkage to bacterial resistance. Environ. Sci. Technol. 49, 6772–6782. doi: 10.1021/acs.est.5b00729, PMID: 25961663

[ref51] Zheng D. Yin G. Liu M. Hou L. Yang Y. van Boeckel T. P.. (2022). Global biogeography and projection of soil antibiotic resistance genes. Sci. Adv. 8:eabq8015. doi: 10.1126/sciadv.abq8015 36383677 PMC9668297

