# Supplementary material for: Antibiotic resistance profiles of gut microbiota across various primate species in Guangxi
Source: Front Microbiol. 2023 Dec 14;14:1309709. doi: 10.3389/fmicb.2023.1309709 (PMC10753005; doi:10.3389/fmicb.2023.1309709)
Supplement: Supplementary file 1 [file Presentation_1.pdf]

Supporting information for Antibiotic Resistance Profiles of Gut Microbiota Across Various Primate Species in Guangxi

Hongli Huang[1#], Xianwu Pang[2#], Tengcheng Que[3,4,5], Panyu Chen[5], Shousheng Li[5], Aiqiong Wu[5], Meihong He[5], Hong Qiu[6], Yanling Hu[6, 7, 8*]

*Correspondence: ylhupost@163.com

[1]Clinical Biological Specimen Bank, Discipline Construction Office, The First Affiliated Hospital of Guangxi Medical University, Nanning, Guangxi, 530021, China.

[2]Guangxi Zhuang Autonomous Region Center for Disease Prevention and Control, Nanning, Guangxi, 530028, China.

[3]Faculty of Data Science, City University of Macau, Macau, 999878, China

[4]Right river national medical college, Baise, Guangxi, 533000, China

[5]Guangxi Zhuang Autonomous Region Terrestrial Wildlife Course Research and Epidemic Diseases Monitor Center, Nanning, Guangxi, 530028, China

[6]Life Sciences Institute, Guangxi Medical University, Nanning, Guangxi, 530021, China.

[7]Department of Biochemistry and Molecular Biology, School of Pre-Clinical Medicine, Guangxi Medical University, Nanning, Guangxi, 530021, China.

[8]Center for Genomic and Personalized Medicine, Guangxi key Laboratory for Genomic and Personalized Medicine, Guangxi Collaborative Innovation Center for Genomic and Personalized Medicine, Guangxi Medical University, Nanning, Guangxi, 530021, China.

Figure S1 Geographic distribution of nine NHP species.

Figure S2 Krona species annotation of ten primate species. (A) *Homo sapiens*, (B) *T. leucocephalus*, (C) *M. mulatta*, (D) *N. coucang*, (E) *N. pygmaeus*, (F) *Lemur catta*, (G) *T. francoisi* (Nanning Zoo), (H) *T. francoisi* (Wuzhou), (I) *T. cristatus*, (J) *Hylobatidae*, (K) *Papio*.

Figure S3 Gut microbiome composition variation among different primate individuals.

Figure S4 Taxonomic features discriminating gut microbiomes among different primate individuals (LDA score > 4.0).

Figure S5 ARG distribution across all samples.

Figure S6 Comparative analysis of ARG types among ten primate species. (A) *T. leucocephalus*, (B) *M. mulatta*, (C) *N. coucang*, (D) *N. pygmaeus*, (E) *Lemur catta*, (F) *T. Francoisi* (Nanning Zoo), (G) *T. francoisi* (Wuzhou), (H) *T. cristatus*, (I) *Hylobatidae*, (J) *Papio*, (K) *Homo sapiens*.

Figure S7 Comparative analysis of top four abundant resistance types (Tetracycline, MLS, Beta-lactam, Multidrug) among different groups.

Figure S8 Variation in ARG subtypes among different primate individuals.

Figure S9 PCA plot showing the similarities of the ARG subtype composition among different primate groups.

Figure S10 Comparative analysis of ARGs abundance in *T. francoisi* from two different locations (Wuzhou langur breeding and research center and Nanning Zoo)

Table S1 Sample information used in the study.

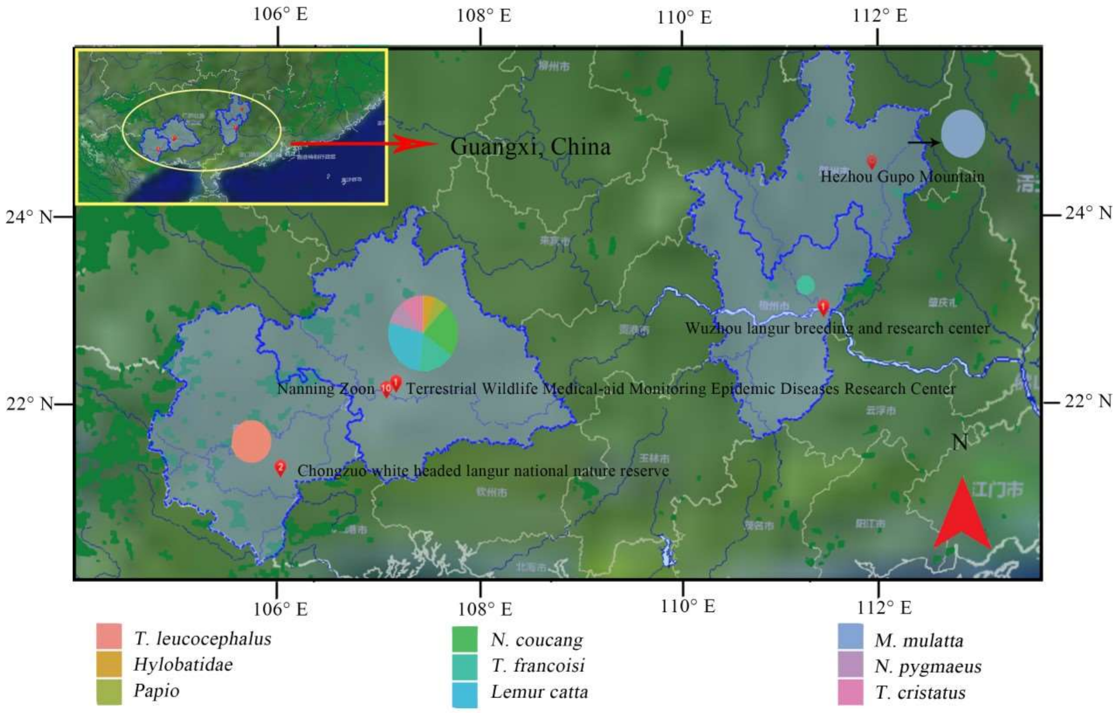

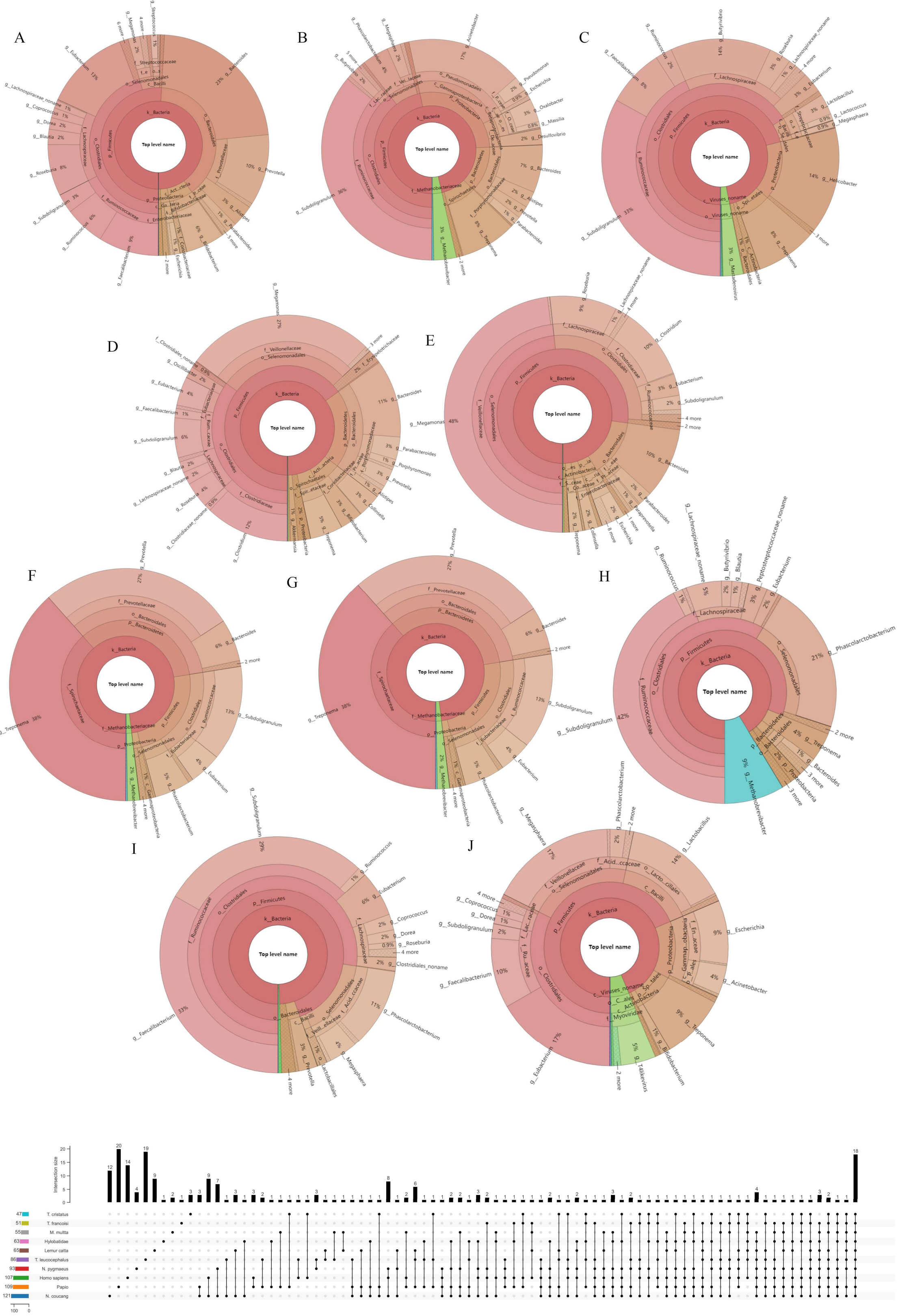
A
B
C
D
E
F
G
H
I
J
Top level name
k__Bacteria
Intersection size
47 T. cristatus
51 T. francoisi
55 M. multta
63 Hylobatidae
65 Lemur catta
86 T. leucocephalus
93 N. pygmaeus
107 Homo sapiens
109 Papio
121 N. coucang
100
0

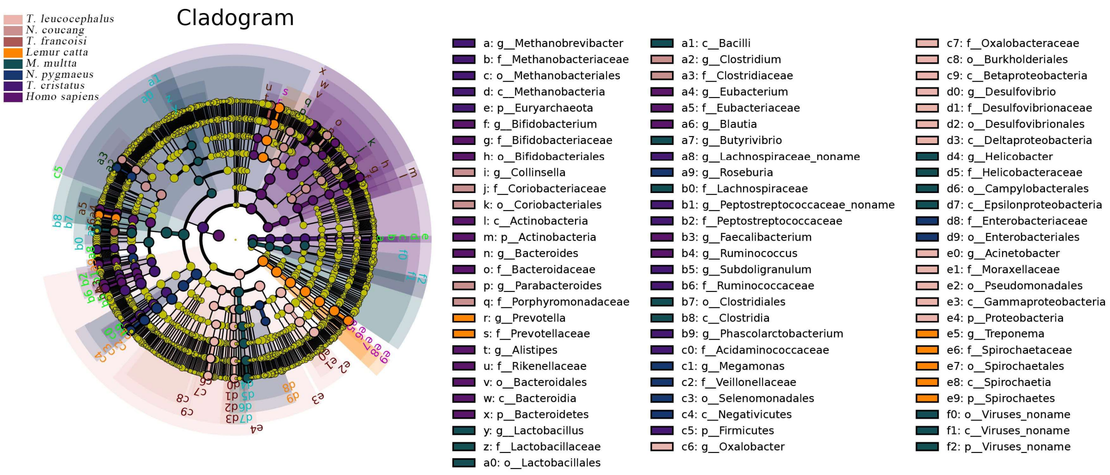
Cladogram
T. leucocephalus
N. coucang
T. francoisi
Lemur catta
M. multta
N. pygmaeus
T. cristatus
Homo sapiens
a: g__Methanobrevibacter
b: f__Methanobacteriaceae
c: o__Methanobacteriales
d: c__Methanobacteria
e: p__Euryarchaeota
f: g__Bifidobacterium
g: f__Bifidobacteriaceae
h: o__Bifidobacteriales
i: g__Collinsella
j: f__Coriobacteriaceae
k: o__Coriobacteriales
l: c__Actinobacteria
m: p__Actinobacteria
n: g__Bacteroides
o: f__Bacteroidaceae
p: g__Parabacteroides
q: f__Porphyromonadaceae
r: g__Prevotella
s: f__Prevotellaceae
t: g__Alistipes
u: f__Rikenellaceae
v: o__Bacteroidales
w: c__Bacteroidia
x: p__Bacteroidetes
y: g__Lactobacillus
z: f__Lactobacillaceae
a0: o__Lactobacillales
a1: c__Bacilli
a2: g__Clostridium
a3: f__Clostridiaceae
a4: g__Eubacterium
a5: f__Eubacteriaceae
a6: g__Blautia
a7: g__Butyrivibrio
a8: g__Lachnospiraceae_noname
a9: g__Roseburia
b0: f__Lachnospiraceae
b1: g__Peptostreptococcaceae_noname
b2: f__Peptostreptococcaceae
b3: g__Faecalibacterium
b4: g__Ruminococcus
b5: g__Subdoligranulum
b6: f__Ruminococcaceae
b7: o__Clostridiales
b8: c__Clostridia
b9: g__Phascolarctobacterium
c0: f__Acidaminococcaceae
c1: g__Megamonas
c2: f__Veillonellaceae
c3: o__Selenomonadales
c4: c__Negativicutes
c5: p__Firmicutes
c6: g__Oxalobacter
c7: f__Oxalobacteraceae
c8: o__Burkholderiales
c9: c__Betaproteobacteria
d0: g__Desulfovibrio
d1: f__Desulfovibrionaceae
d2: o__Desulfovibrionales
d3: c__Deltaproteobacteria
d4: g__Helicobacter
d5: f__Helicobacteraceae
d6: o__Campylobacterales
d7: c__Epsilonproteobacteria
d8: f__Enterobacteriaceae
d9: o__Enterobacteriales
e0: g__Acinetobacter
e1: f__Moraxellaceae
e2: o__Pseudomonadales
e3: c__Gammaproteobacteria
e4: p__Proteobacteria
e5: g__Treponema
e6: f__Spirochaetaceae
e7: o__Spirochaetales
e8: c__Spirochaetia
e9: p__Spirochaetes
f0: o__Viruses_noname
f1: c__Viruses_noname
f2: p__Viruses_noname

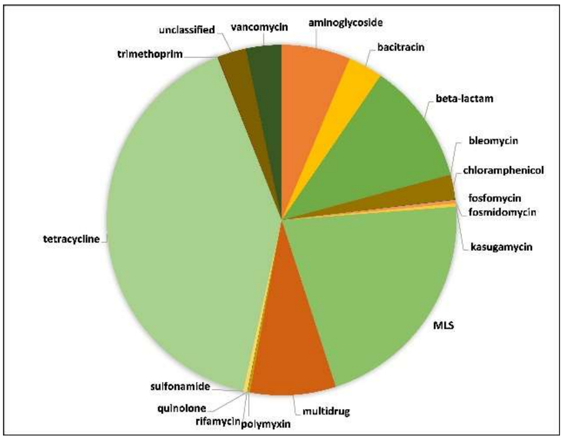
unclassified
vancomycin
aminoglycoside
trimethoprim
bacitracin
beta-lactam
bleomycin
chloramphenicol
fosfomycin
fosmidomycin
tetracycline
kasugamycin
MLS
sulfonamide
quinolone
rifamycin
polymyxin
multidrug

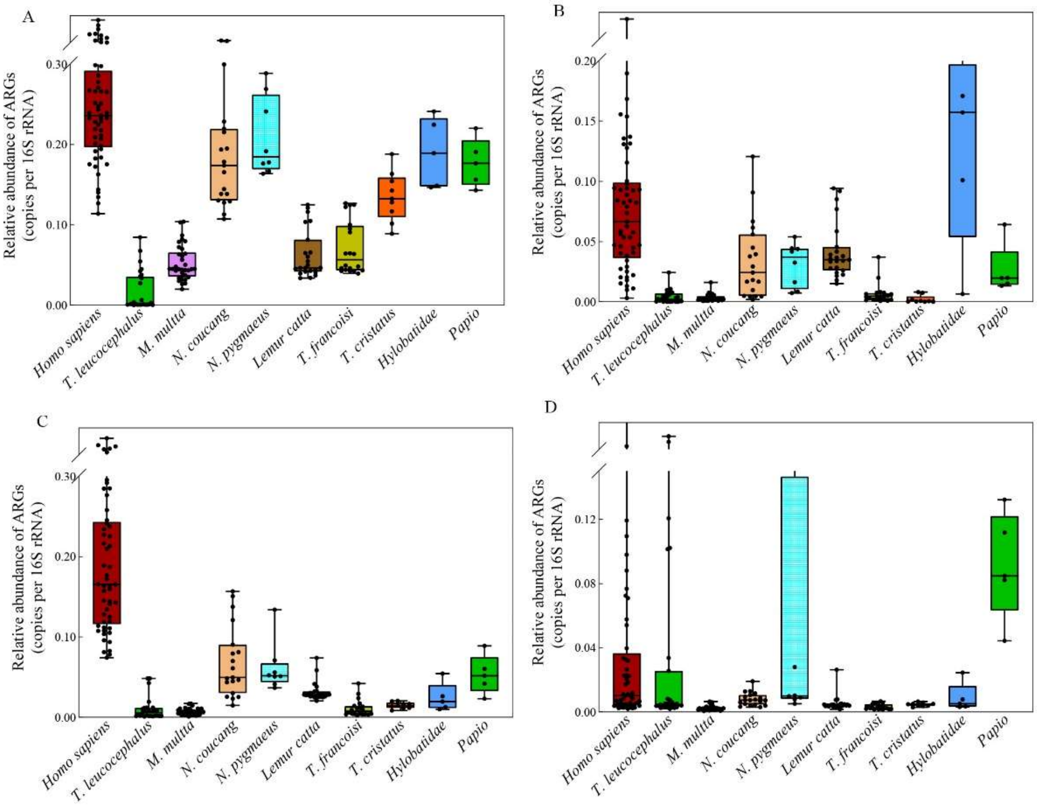
A
B
C
D
Relative abundance of ARGs (copies per 16S rRNA)
Homo sapiens
T. leucocephalus
M. multta
N. coucang
N. pygmaeus
Lemur catta
T. francoisi
T. cristatus
Hylobatidae
Papio

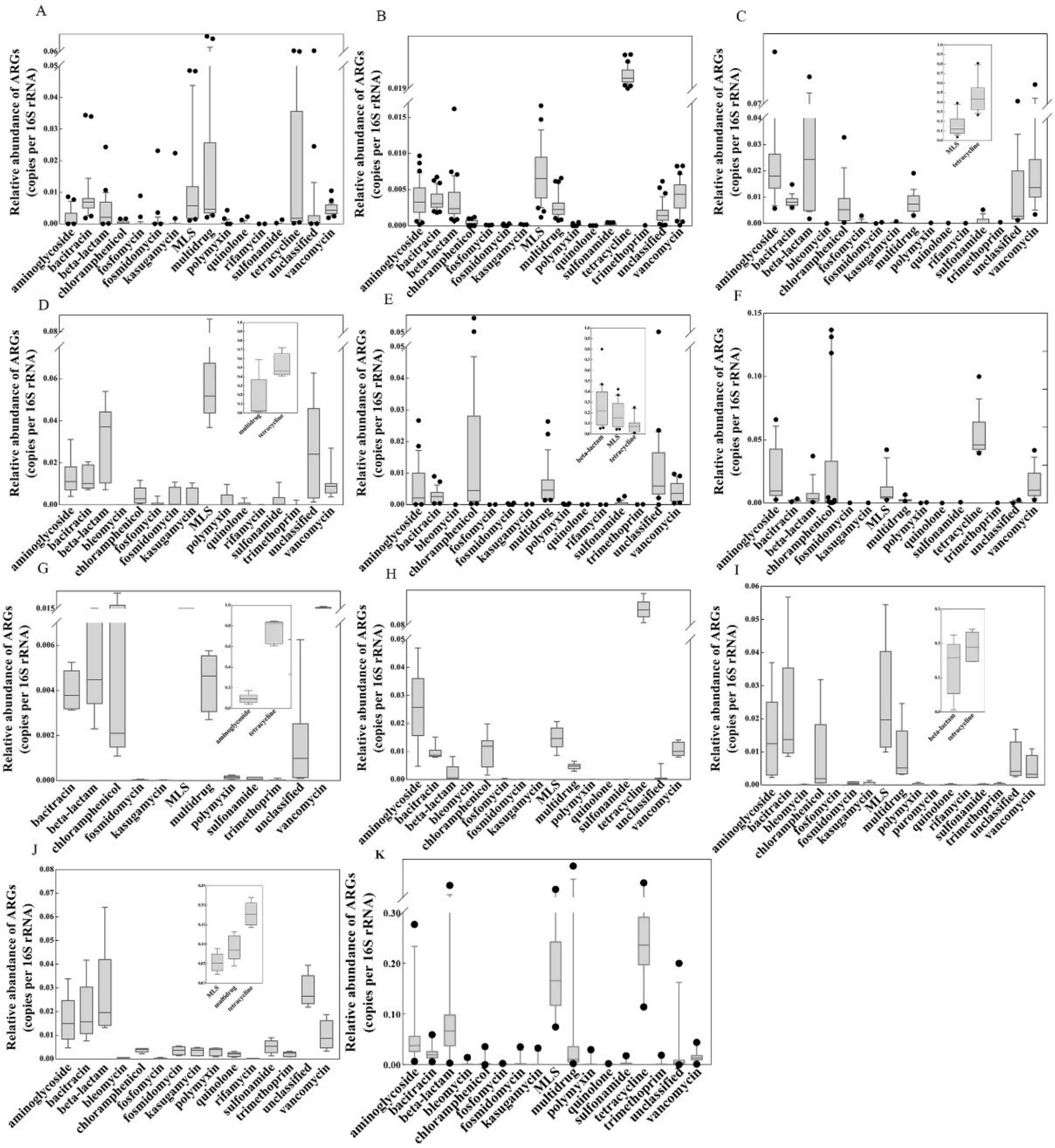
A
B
C
D
E
F
G
H
I
J
K
Relative abundance of ARGs (copies per 16S rRNA)
aminoglycoside
bacitracin
beta-lactam
bleomycin
chloramphenicol
fosfomycin
fosmidomycin
kasugamycin
MLS
multidrug
polymyxin
quinolone
rifamycin
sulfonamide
tetracycline
trimethoprim
unclassified
vancomycin

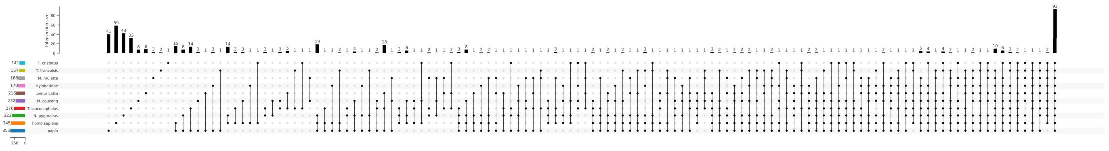

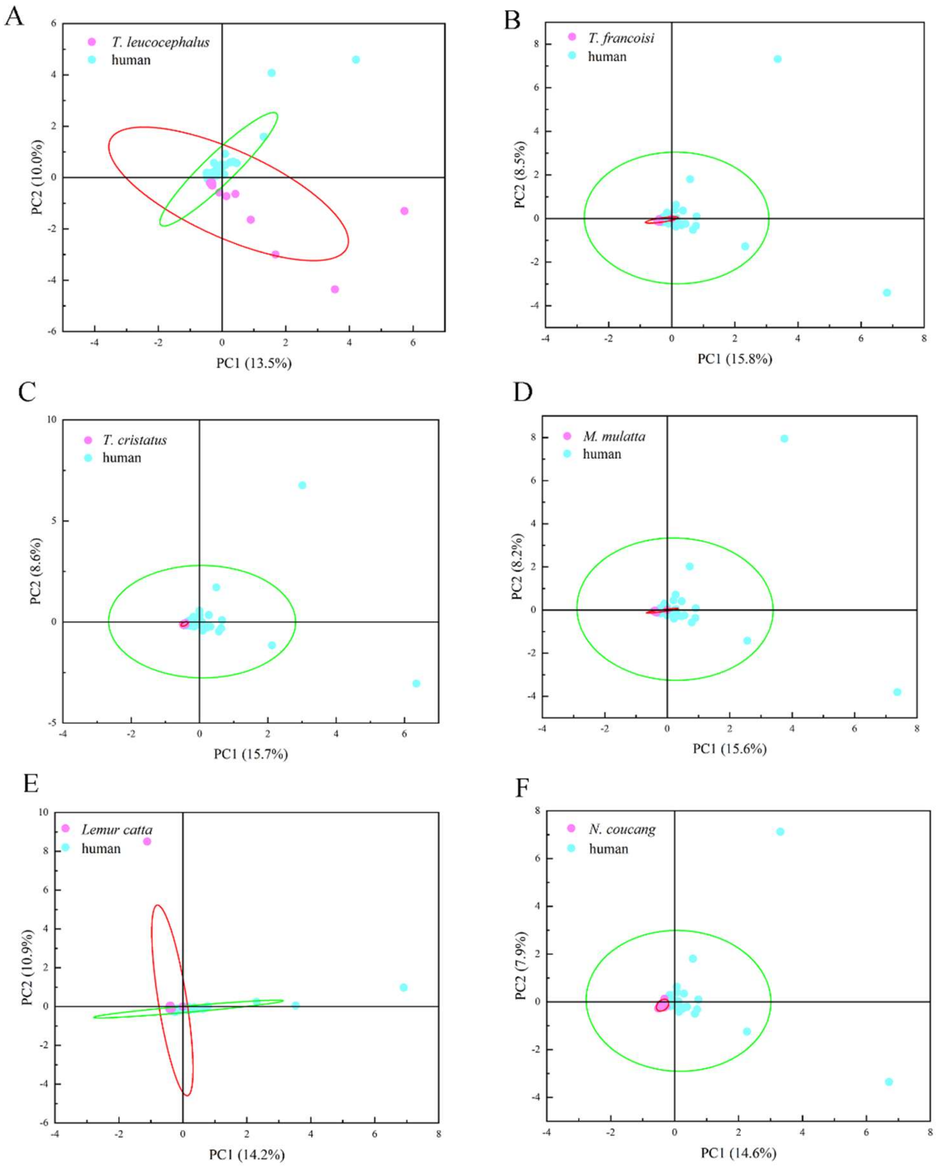
A
T. leucocephalus
human
PC2 (10.0%)
PC1 (13.5%)
B
T. francoisi
human
PC2 (8.5%)
PC1 (15.8%)
C
T. cristatus
human
PC2 (8.6%)
PC1 (15.7%)
D
M. mulatta
human
PC2 (8.2%)
PC1 (15.6%)
E
Lemur catta
human
PC2 (10.9%)
PC1 (14.2%)
F
N. coucang
human
PC2 (7.9%)
PC1 (14.6%)

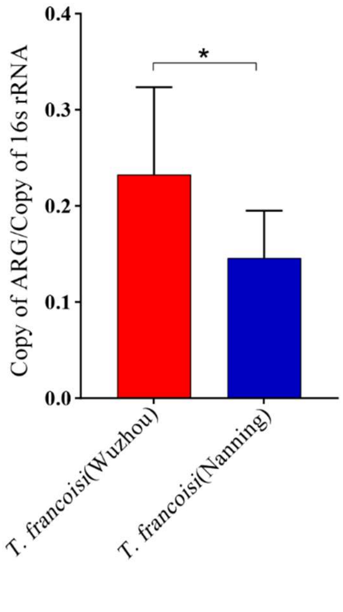
Copy of ARG/Copy of 16s rRNA
0.0
0.1
0.2
0.3
0.4
*
T. francoisi(Wuzhou)
T. francoisi(Nanning)

Species	Numbers	Location	Diet	Living style
Homo sapiens	53	China	--	--
Trachypithecus leucocephalus (*T. leucocephalus*)	1	Nanning	phytophagous	captive
Trachypithecus leucocephalus (*T. leucocephalus*)	26	Chongzuo	phytophagous	wild
Hylobatidae	5	Nanning	omnivorous	captive
Papio	5	Nanning (Zoo)	omnivorous	captive
Macaca multta (*M. multta*)	34	Hezhou	omnivorous	wild
Trachypithecus francoisi (*T. francoisi*)	6	Wuzhou	phytophagous	captive
Trachypithecus francoisi (*T. francoisi*)	14	Nanning (Zoo)	phytophagous	captive
Lemur catta	23	Nanning (Zoo)	phytophagous	captive
Nycticebus pygmaeus (*N. pygmaeus*)	8	Nanning	omnivorous	captive
Trachypithecus cristatus (*T. cristatus*)	9	Nanning (Zoo)	phytophagous	captive
Nycticebus coucang (*N. coucang*)	19	Nanning	omnivorous	captive
